# PAI-1, the Plasminogen System, and Skeletal Muscle

**DOI:** 10.3390/ijms21197066

**Published:** 2020-09-25

**Authors:** Fasih Ahmad Rahman, Matthew Paul Krause

**Affiliations:** 1 Department of Kinesiology, University of Waterloo, Waterloo, ON N2L 3G5, Canada; fasih.rahman@uwaterloo.ca; 2 Department of Kinesiology, University of Windsor, Windsor, ON N9B 3P4, Canada

**Keywords:** plasminogen activator inhibitor-1, plasminogen system, skeletal muscle, pathophysiology, regeneration, muscular dystrophy, diabetes, aging, exercise, therapeutics

## Abstract

The plasminogen system is a critical proteolytic system responsible for the remodeling of the extracellular matrix (ECM). The master regulator of the plasminogen system, plasminogen activator inhibitor-1 (PAI-1), has been implicated for its role in exacerbating various disease states not only through the accumulation of ECM (i.e., fibrosis) but also its role in altering cell fate/behaviour. Examination of PAI-1 has extended through various tissues and cell-types with recent investigations showing its presence in skeletal muscle. In skeletal muscle, the role of this protein has been implicated throughout the regeneration process, and in skeletal muscle pathologies (muscular dystrophy, diabetes, and aging-driven pathology). Needless to say, the complete function of this protein in skeletal muscle has yet to be fully elucidated. Given the importance of skeletal muscle in maintaining overall health and quality of life, it is critical to understand the alterations—particularly in PAI-1—that occur to negatively impact this organ. Thus, we provide a comprehensive review of the importance of PAI-1 in skeletal muscle health and function. We aim to shed light on the relevance of this protein in skeletal muscle and propose potential therapeutic approaches to aid in the maintenance of skeletal muscle health.

## 1. Introduction

The plasminogen system is a proteolytic system responsible for the degradation of several proteins, most notably, fibrin. The initial study identifying key features of this process particularly during the dissolution of blood clots was first published in 1893 by a French physiologist named Albert Dastre [1]. Dastre coined the term “fibrinolysis” and it was not until the mid-twentieth century where major aspects of this process were revealed, including the identification of the circulating latent zymogen, plasminogen, and its active form, plasmin [1,2,3,4,5,6,7]. A role for the plasminogen system in modulating extracellular matrix (ECM) structure has since been identified in several tissues. Plasmin is capable of directly degrading fibrin or activating downstream proteases to degrade structural proteins of the ECM. Plasminogen is activated by plasminogen activators (PAs): tissue-type PA (tPA) and urokinase-type PA (uPA). It was not until the 1980s when the major regulator of the plasminogen system, plasminogen activator inhibitor-1 (PAI-1), was identified [8,9]. PAI-1 tightly regulates the activity of tPA and uPA and as a result prevents a constant state of fibrinolysis [8,9]. Perhaps reflecting the diversity of biological roles played by PAI-1, including regulation of cell death [10,11], senescence [12,13], and inflammation [14,15], PAI-1 has been identified as a bona fide marker in several pathological conditions. Given that PAI-1 is present within the circulation under normal and pathological conditions, it is unsurprising that this protein is capable of affecting virtually all tissues/organs in the body. For instance, high levels of PAI-1 have been implicated in cancer [16,17,18,19,20], cardiovascular disease [21,22,23], diabetic complications [23,24], renal disease [25,26], musculoskeletal disorders [27,28,29,30], and tissue fibrosis [31,32,33]. In skeletal muscle, PAI-1 plays critical roles in response to skeletal muscle injury and in myopathic conditions [27,28,29,34,35], further highlighting the importance of the plasminogen system and ECM remodeling to skeletal muscle. Indeed, several studies have identified the importance of plasminogen system function but have yet to fully elucidate a role for PAI-1 in skeletal muscle [35,36,37,38,39,40], although recent evidence suggests that skeletal muscle is also a site of PAI-1 synthesis [29,41,42]. Control of PAI-1 levels in the circulation is of clinical importance and while studies generally indicate that circulating PAI-1 can be controlled with exercise [43,44,45,46,47,48,49,50,51,52,53,54,55,56,57,58,59,60] or through the administration of pharmacological PAI-1 inhibitors [17,22,61,62,63,64,65,66,67,68,69,70,71]. This review will summarize the biology of PAI-1 and the plasminogen system as it relates to overall tissue function and ECM remodeling, highlight the role of PAI-1 in skeletal muscle, and shed light on potential therapeutic strategies to reduce or normalize PAI-1 levels in an attempt to improve muscle and overall health.

## 2. Basic Biology of PAI-1 and the Plasminogen System

### 2.1. Structure and Function of PAI-1

**Figure 1:**
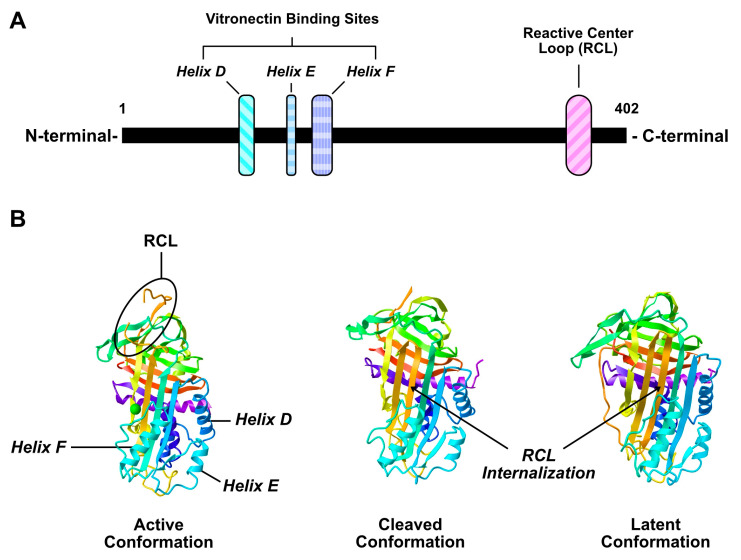
Illustration of plasminogen activator inhibitor-1 (PAI-1) linear (**A**) and crystalline (**B**) structure. Crystalline structure adapted from [101,102].

PAI-1 is secreted as a single chain molecule that contains two distinct interactive domains; (1) a reactive center loop (RCL), (2) a flexible joint region with helix D (hD), helix E (hE), and helix F (hF) binding sites (Figure 1A) [16]. The RCL domain is the primary site for uPA/tPA binding and contains a P1-P1′ peptide bond that interacts with these proteases. Upon cleavage of the P1-P1′ bond, PAI-1/PA molecules form an irreversible complex resulting in the inactivation of PA, and partial internalization of the RCL domain (Figure 1B) [72,73,74]. uPA and tPA may also cleave PAI-1 without forming an irreversible complex [75]. In this instance, PAI-1 is still capable of interacting with other proteins with its helix domains, but its ability to inhibit uPA/tPA is abolished [75].

**Figure 2:**
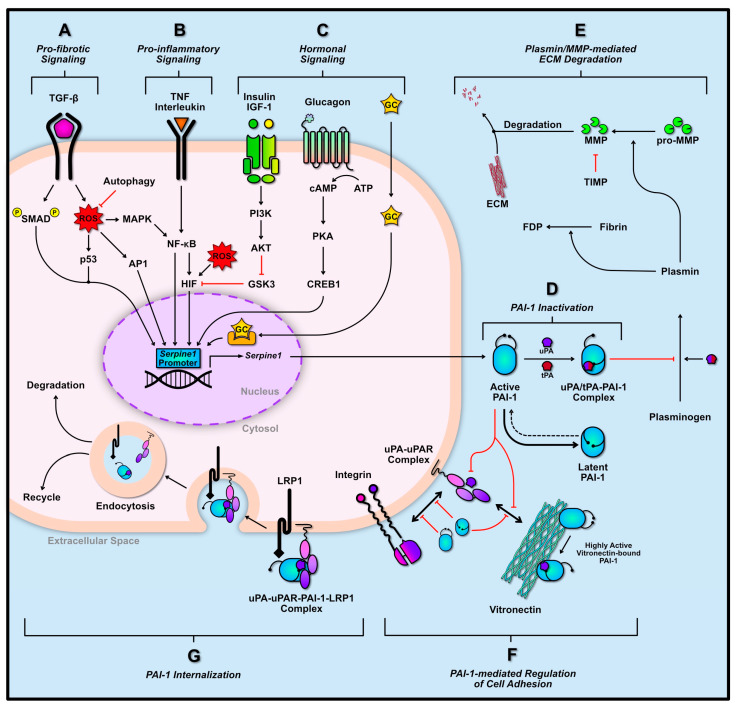
*Serpine1* transcriptional regulation and PAI-1 function. *Serpine1* can be transcribed through several signaling cascades including pro-fibrogenic (**A**), pro-inflammatory (**B**), and pro-growth/hormonal signaling cascades (**C**). Once transcribed, PAI-1 is secreted in its active form into the extracellular space where it can inhibit urokinase-type PA (uPA)/tissue-type PA (tPA), and thus inhibit downstream extracellular matrix (ECM) degradation by preventing matrix metalloproteinase (MMP) activation (**D**,**E**). Conversely, PAI-1 may be rapidly converted to its more stable latent state. The active and latent PAI-1 molecules can interact with uPA/uPA receptor (uPAR) and integrins to diminish cell adhesion to vitronectin (**F**). Vitronectin-bound PAI-1 prevents its premature conversion to its latent state and improves its binding affinity to uPA/tPA. PAI-1 may also be internalized by the cell, through its interaction with lipoprotein receptor-related protein 1 (LRP1) and uPA/uPAR, ultimately leading to its degradation or recycling (**G**). Solid black arrows indicate activation. Dotted black lines indicate potential yet unfavorable pathways. Red bars indicate inhibition or blockage. Two-way arrows indicate interaction between proteins.

Binding of the flexible domain of PAI-1 (hD, hE, and hF sites) to vitronectin prevents adjacent binding of vitronectin with integrin and stabilizes PAI-1 in its active form while increasing its binding affinity with uPA/tPA 200-fold (Figure 2F) [76,77,78]. Furthermore, PAI-1 can detach cells through the interaction with uPA bound to its receptor (uPAR). In the absence of PAI-1, uPA bound to uPAR results in a conformational change that increases uPAR affinity for vitronectin or other integrins, and thus, maintain cell adhesion [79,80,81,82]. However, PAI-1 binding to uPA-uPAR can disrupt this interaction with vitronectin or integrin, reducing adhesion/migration of cells (Figure 1F) [79,80,81,82,83,84]. PAI-1 may also interact directly with the somatomedin B domain on vitronectin with greater affinity than uPAR, thus, further diminishing cell adhesion [85]. Furthermore, PAI-1 bound to the uPA-uPAR complex can interact with lipoprotein receptor-related protein 1 (LRP1) found on the cell membrane via its hD and hE domains, and trigger endocytosis of the uPAR complex; resulting in reduced levels of uPA and uPAR (Figure 2G) [76,86]. Together, the lack of uPA/uPAR activity, and diminished cell adhesion and migration may potentiate apoptosis of cells [87].

Thorough assessment of PAI-1 structure has also revealed that this protein is secreted from cells in its active form, however this form is short lived. The typical half-life of active PAI-1 is between 1–2 h before it is spontaneously converted to its highly stable latent (partially inactive) form [88,89,90,91]. Similar to the cleavage of the P1-P1′ peptide bond by plasminogen activators resulting in internalization of the RCL domain (Figure 2D), this phenomenon can occur spontaneously without the cleavage of the P1-P1′ bond, and this conformation may serve as a regulatory mechanism to prevent prolonged anti-fibrinolytic action of PAI-1 [75]. Nonetheless, the latent form can be reactivated by denaturing and refolding, although this event may not be physiologically relevant [92,93]. Similar to the cleaved form of PAI-1, latent PAI-1 can interact with cell surface receptors or ECM molecules via its helix domains or it may bind directly to fibrin as a result of this new conformation to inhibit tPA-induced degradation (Figure 2D,F) [94].

As the master regulator of the plasminogen system, PAI-1 plays an important role in ECM remodeling through the modulation of matrix metalloproteinase (MMP) activity. Although PAI-1 does not interact with MMP directly, its upstream inhibitory role on plasmin activation diminishes the cleavage-mediated activation of pro-MMP (Figure 2E) [32,95]. Interestingly, plasmin is also capable of inducing increased MMP secretion whereas its zymogen (i.e., plasminogen) can induce increased secretion of PAI-1 [95]. The induction of PAI-1 in this manner may serve as a negative-feedback mechanism to limit plasmin- and MMP-mediated ECM degradation [95]. Tissue inhibitors of metalloproteinases (TIMP)s are typically expressed concurrently with PAI-1 [96,97,98,99]. For example, fibrogenic signaling cascades tend to increase levels of PAI-1 and TIMPs together [98,99,100]. TIMPs directly inhibit MMPs, thereby blocking ECM degradation.

### 2.2. Transcriptional Regulation of PAI-1

PAI-1 is rapidly synthesized and secreted in response to multiple signaling cascades. The transcriptional regulation of which has been largely investigated. PAI-1 is expressed in vasculature (endothelial and smooth muscle cells), immune cells, heart, liver, kidney, adipose tissue, as well as some cancer cell types [18,20,103]. Skeletal muscle also appears to express PAI-1, at least during regeneration, suggesting that PAI-1 plays a role in modulating skeletal muscle ECM [29,41,42] (GEO dataset: GDS234; Reference series GSE469). Regardless of tissue origin, the transduction of *Serpine1* (i.e., gene encoding for PAI-1) remains similar across most if not all tissue types. This section will highlight the major contributors to *Serpine1* transduction in three categories: (1) pro-fibrotic signaling, (2) pro-inflammatory signaling, and (3) hormonal signaling (Figure 2A–C).

The pro-fibrotic signaling of transforming growth factor-β (TGF-β) is a major contributor to PAI-1 transduction (Figure 2A). The canonical activation of TGF-β signaling occurs through the binding of TGF-β to its receptor resulting in the phosphorylation and activation of SMAD2/3. Activated SMAD2/3 can associate with SMAD4 and translocate to the nucleus and bind to the *Serpine1* promoter, along with other pro-fibrotic promoter regions [104,105]. The TGF-β cascade has multiple non-canonical pathways as well. These include the elevation of mitochondrial and cytosolic reactive oxygen species (ROS), resulting in the subsequent activation of mitogen-associated protein kinase (MAPK), and nuclear factor kappa B (NF-κB) [106,107,108,109]. In fact, the production of ROS is thought to be a major mediator of TGF-β-mediated *Serpine1* transcription [110]. Elevations in ROS also signal the transduction of cell cycle regulator p53, which alongside SMAD2/3/4, can aid in the transcription of *Serpine1* [111,112]. ROS signaling can also stimulate activator protein-1 (AP-1), and hypoxia-inducing factor-1α (HIF-1α) [113,114,115]. In addition to its regulation of *Serpine1*, TGF-β signaling has been shown to increase the half-life of *Serpine1* mRNA, thereby increasing PAI-1 protein translation [116].

*Serpine1* expression can also be induced in response to characteristic inflammatory signaling including tumor necrosis factors (TNF) and interleukins (IL) (Figure 2B). Binding of TNF to its respective receptor can lead to the direct activation of NF-κB, and subsequent *Serpine1* transcription [117,118,119]. Furthermore, the inhibition of TNFα via adiponectin has also been shown to reduce *Serpine1* expression [120]. IL-signaling demonstrate similar transduction of *Serpine1* to that of TNF. Binding of IL receptors can directly activate NF-κB (e.g., IL-1R), or elevate MAPK levels (e.g., IL-6R) within cells, once again leading to the transcription of *Serpine1* [117,121,122].

Several hormones influence *Serpine1* transcription, including insulin, glucagon, and glucocorticoids (GC) (Figure 2C). Both proinsulin, insulin, and insulin-like growth factor-1 (IGF-1) have been shown to induce *Serpine1* expression [117,123]. This was found to be a result of activation AKT, leading to the inhibition of glycogen synthase kinase-3 (GSK3) [117]. Inactivation of GSK3 prevents the inhibitory phosphorylation of HIF, enabling it to translocate to the nucleus and transcribe *Serpine1* [117]. Binding of glucagon to its G protein coupled receptor stimulates adenylate cyclase and leads to elevated cytosolic concentration of cyclic AMP (cAMP), subsequently activating protein kinase A (PKA). Activated PKA can phosphorylate cAMP-response element binding protein-1 (CREB1), which can translocate to the nucleus and transcribe *Serpine1* [124]. Finally, the translocation of lipid soluble GC into the nucleus and their interaction with nuclear receptors can also mediate *Serpine1* transcription [124].

## 3. Role of PAI-1 Following Skeletal Muscle Damage

**Figure 3:**
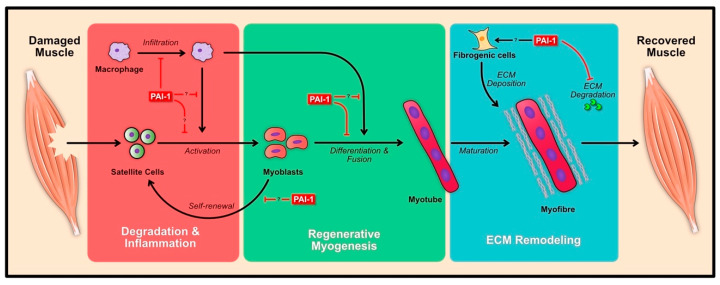
Effects of PAI-1 during skeletal muscle regeneration. Induction of muscle damage results in the swift infiltration of immune cells (macrophages shown), resulting in the degradation of cellular debris and activation of satellite cells to form myoblasts. The activated myoblasts differentiate and fuse to form myotubes, which then mature within their niche to form complete myofibres. PAI-1 has been shown to inhibit the regeneration process at various levels (red bar lines), however the role of PAI-1 relating to satellite cell, myoblast and other cells (immune and fibrogenic cells) during regeneration has yet to be explored. Solid black arrows indicate activation. Red bars indicate inhibition or blockage.

Skeletal muscle is an organ that displays high plasticity in response to damage and is able to repair or regenerate its architecture within a relatively short time span (approx. 10 days) and return it to normal function. Given that contractile events can cause tremendous stress in skeletal muscle, coupled with the fact that physical trauma can severely hinder muscle function, it is critical to understand the nuances of the regeneration process. The regeneration of skeletal muscle can be broadly categorized into three phases: (1) the degeneration and inflammation phase, (2) regenerative myogenesis of muscle stem cell (i.e., satellite cells), and (3) remodeling of the ECM (Figure 3). Each of these phases is characterized by the distinct involvement of multiple cell types, yet there is considerable overlap. For instance, remodeling of the ECM occurs as early as the onset of the degeneration and inflammation phase. Similarly, as the inflammatory phase subsides, myogenesis begins to ramp up. In essence, the balance of the antagonistic roles of PAI-1 and uPA have been implicated during all phases of the regeneration process. Albeit, the intricacies of PAI-1 during skeletal muscle regeneration has yet to be fully elucidated, the known function of PAI-1 and other effectors of the plasminogen system on skeletal muscle regeneration will be highlighted in this section.

### 3.1. Degeneration and Inflammation

Once damaged, skeletal muscle begins to efficiently orchestrate its regenerative response. The first phase involves the degradation of cellular debris and inflammation accompanied by immune cells. Neutrophils are the among the first to arrive at the site of damage within 24 h and begin to degrade debris through phagocytosis [125]. Neutrophils also release chemotactic signals to attract highly important monocytes. Unlike neutrophils, monocytes or macrophages are essential for regeneration, the lack or aberrant expression of this cell type has been shown to impair the regenerative response in muscle. Infiltrated monocytes can differentiate to classical M1 macrophage and take over the bulk degradation of cellular debris. Afterwards, these cells may further differentiate into alternative M2 macrophages, releasing anti-inflammatory signals that ease the degradation of tissue and signal for its reconstruction.

The generalized role of the plasminogen system has been shown to be important during skeletal muscle regeneration with multiple proteins playing a part in this coordinated response. For instance, the knockout (KO) of *Plg* (i.e., gene encoding for plasminogen) has been shown to severely impair regeneration after glycerol-induced muscle damage [37]. It was shown that in *Plg* KO muscle, the infiltration of neutrophils and macrophages was diminished 2 days post injury [37]. However, macrophage infiltration and regeneration were rescued in these KO animals through the systemic treatment of fibrinogenolytic agent, ancrod [37]. It was also noted that plasminogen activation was highly dependent upon uPA activity but not tPA [37]. Thus, both plasmin and uPA are essential for muscle regeneration.

Following cardiotoxin (CTX)-induced muscle damage, the activity of uPA and tPA increases; with a greater magnitude of increase in uPA [28]. In *Serpine1* KO animals, uPA levels were elevated from 1 day and remained elevated up to 5 days post-CTX [28]. Interestingly, when investigating muscle force production and presence of necrotic area, *Serpine1* KO animal were found to recovery muscle strength (i.e., force), and demonstrated rapid necrotic area clearance compared to wild-type (WT) animals [28]. Furthermore, muscle force and regeneration were significantly impaired in *Plau* (i.e., gene encoding for uPA) KO animals, suggesting that uPA activity is needed for regeneration and PAI-1 impairs regeneration. *Plau* KO but not *Plaur* (i.e., gene encoding for uPA receptor) KO animals also displayed impaired macrophage infiltration while neutrophil infiltration remained unchanged [28,126]. Furthermore, macrophage-specific secretion of uPA was found to be important in muscle regeneration by modulating fibrin degradation, and promotion of ECM breakdown [127]. In contrast, *Serpine1* KO animals were found to have elevated macrophage numbers from 3 days to 5 days post-CTX [28]. Together these data suggest that unbound/soluble uPA activity is needed for muscle regeneration, and PAI-1 can negatively impact its role in regeneration. In fact, in diabetic and aged muscle, where PAI-1 levels are elevated, aberrant macrophage infiltration has also been observed [27,29]. Interestingly, however, the chemical inhibition of PAI-1 by tiplaxtinin (PAI-039) was found to rescue early impairments in regeneration in diabetic muscle, particularly in the necrotic regions, and likely by facilitating macrophage infiltration [27,128]. It is unclear whether muscle tissue PAI-1 levels interfere with the function of infiltrated macrophages, specifically in their ability to activate muscle stem cells.

### 3.2. Regenerative Myogenesis

Indeed, the involvement of immune cells, namely macrophages, is important in initiating skeletal muscle regeneration. However, the cells that are central for the regeneration of muscle fibres are satellite cells. Upon activation, satellite cells undergo a several rounds of division, migrate to the site of damage, differentiate to form myotubes, and fuse together to form new muscle fibres [129,130]. The differential expression of myogenic regulatory factors (MRFs) is essential for the progression of satellite cells to form de novo myofibres. Myogenic determination protein 1 (MYOD1), and myogenin (MYOG) are among the two important factors that have been associated with several functions during the regeneration process. Naturally, an impairment in any of these key events or aberrant expression of these MRFs during myogenesis results in impaired muscle regeneration; and this is often accompanied by fibrosis.

The aforementioned studies [126,128,131] have also briefly investigated PAI-1 on satellite cell function. *Serpine1* KO animals were found to induce greater MYOD1 levels, embryonic myosin heavy chain content, and centrally located nuclei; indicating increased regeneration [28]. Interestingly, *Serpine1* KO animals were able to regenerate muscle at a higher rate compared to WT [28]. A similar finding was observed in diabetic muscle, where the chemical inhibition of PAI-1 was found to improve satellite cell activation in the necrotic regions [128]. This mechanism of this phenomena was proposed to be a result of the activity of the downstream matrix metalloproteinase-9 (MMP-9), likely originating from macrophages [29,127,128]. Studies have also demonstrated that active uPA and its function to cleave plasminogen to form plasmin is required for myoblast differentiation and myotube formation [37,38,40]. Thus, the impairment in uPA activity or the KO of *Plg* entirely has negative consequences on myogenesis. Contrary to these findings, a recent study has shown that PAI-1 may not affect myoblast differentiation per se, rather PAI-1 may impair development of fused myotubes. This study showed that the administration of PAI-1 (i.e., gain of function) or the knockdown of *Serpine1* using siRNA had no effect on protein synthesis/AKT signaling, nor did it affect differentiation markers (MYOD1, MYOG, and myosin) [132]. Instead, PAI-1 was found to reduce IGF-1 protein levels (a critical growth factor) within muscle [132], however, further investigation is needed as to whether PAI-1 is associated with morphological changes (length, width, number of nuclei, etc.) of newly formed myotubes.

The role of uPA/PAI-1 balance in mediating cell migration has also been studied in myoblasts. In most instances, the activators of PAI-1 have also been demonstrated to reduce *Plau* mRNA expression. For example, IL-17 treatment of C2C12 myoblasts and subsequent MAPK14 activation was found to reduce *Plau* mRNA and protein expression in a dose dependent manner [121]. In other words, high levels of interleukin-mediated stress signaling in myoblasts suppresses *Plau* expression, and thus impairs fibrinolysis. Both PAI-1 and IL-17 treated cells displayed reduced differentiation and migration in a similar manner [121]. Direct chemical inhibition of uPA activity has also been shown to reduce primary and C2C12 myoblast migration, however, uPAR was found to be dispensable during myogenesis [121,133]. Impaired myoblast migration in *Plau* deficient or PAI-1 treated cells is consistent with in vivo findings demonstrating poor macrophage infiltration [28,29,128]. It may be possible that the muscle tissue-specific PAI-1 levels are limiting cell migration, and thus, leading to poor regeneration. Additionally, the effect of PAI-1 on satellite cell self-renewal has yet to be investigated.

### 3.3. ECM Remodeling

The remodeling of the ECM occurs at every stage of the regeneration process but varies in its magnitude. ECM plasticity is required for the infiltration of immune cells during the early stages of the degeneration/inflammation phase. ECM turnover is also required for the migration of satellite cells and formation of new scaffolding material for the newly formed myotubes to situate themselves within. Indeed, the inhibitory effect of PAI-1 on plasminogen activation plays an important role in ECM remodeling during regeneration, however, the effect of the downstream proteases of the plasminogen system should not be dismissed. Moreover, the genetic ablation or inhibition of plasmin, uPA, and matrix metalloproteinases (MMPs) impairs muscle regeneration.

Several members of the MMP family have been shown to be involved in the remodeling of muscle following damage. MMP-2 and MMP-9, two gelatinases, are among the best characterized. MMP-2 is constitutively expressed in muscle whereas MMP-9 is induced as early as 1 day following CTX-injury [134,135]. The secretion and activity of MMP-9 early during the regeneration process necessitates the need to remove excess ECM material to enable efficient immune cell infiltration into the damage regions [27,29,134,136,137]. Further investigations of MMP-9 have elucidated its colocalization with macrophages early in the regeneration process [29]. In diabetic plasma and aged muscles, where PAI-1 levels are elevated, reduced MMP-9 levels are observed, and they coincide with ECM accumulation, poor satellite cell activation and muscle regeneration [29,128,134]. Under these conditions, PAI-1 is likely inhibiting the activation of MMPs through the inhibition of uPA and plasmin. PAI-1 may also be interacting with fibrogenic cells within the damaged tissue to exacerbate ECM deposition.

C2C12 cells have been established to migrate at a faster rate than primary myoblasts [138]. In the presence of MMP inhibitors, C2C12 migration is diminished after transplantation [138]. In vitro experiments have shown that C2C12 cells fail to express MMP-9 but differentially express MMP-2 during differentiation and is needed for migration [134,138,139]. Activation of MMP-2 via membrane type-I MMP (MT1-MMP) and overexpression of *Mmp2* in myoblasts was found to increase primary myoblast migration [138]. Transplantation of C2C12 cells alongside MMP-1 was also found to improve graft efficiency and muscle regeneration through the remodeling of the ECM [140,141,142]. Considering the active involvement of MMPs in grafting success, it is likely that the selective targeting and transient inhibition of PAI-1 during skeletal muscle grafting would improve engraftment success. Nonetheless, MMP-13 has also been shown to increase throughout the regeneration process and aid in the migration of satellite cells [135]. Surprisingly, MMP-13 overexpression was found to increase MYOG levels in C2C12 cells, yet it did not impact myoblast fusion and is dispensable in resting muscle [135,143].

As myoblasts/satellite cells progress through into the final stages myogenesis—differentiation and fusion—the level of MMPs typically drops and the expression of TIMPs increases [136,139]. Myoblasts typically do not express TIMP during the early stages of differentiation—likely to maximize MMP activity. In fact, the genetic overexpression of *Timp2* in myoblasts prevents myotube formation, whereas the gradual increase in TIMP-1 in myotubes is associated with their maturation [136,139]. Interestingly, the lack of colocalization of TIMP-2 and MMPs in C2C12 myoblasts may suggest the alternative role (i.e., non-ECM-related roles) of these proteins during differentiation [139]; however, this has yet to be fully elucidated.

## 4. Pathological Role of PAI-1 in Skeletal Muscle

### 4.1. Muscular Dystrophy

**Figure 4:**
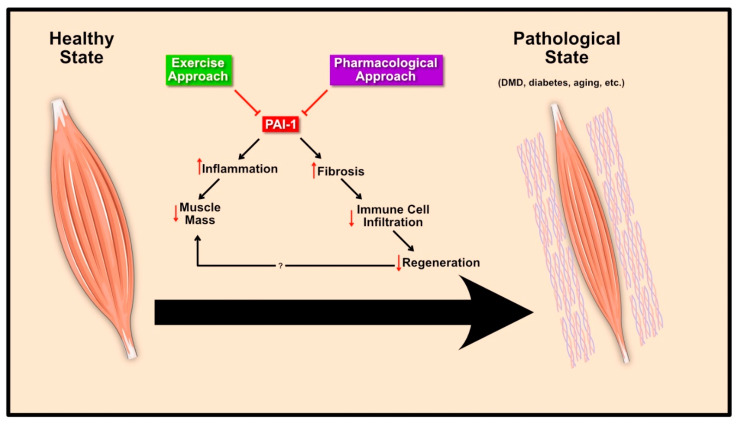
PAI-1 in muscle pathology and therapeutics. Excessive PAI-1 levels lead to increased fibrosis and are associated with inflammation. In skeletal muscle, this results in impaired regeneration and reduced muscle mass in various pathological states. Exercise and pharmacological agents reduce circulating PAI-1 levels and serve as a therapeutic approach to normalize PAI-1 levels and improve skeletal muscle health.

Muscular dystrophy is a class of disease that leads to progressive muscle loss and weakness. Duchenne muscle dystrophy (DMD) is one of the most common forms of X-linked lethal diseases, known to affect almost 1 in 3000 males [144]. This disease originates from a mutation in the gene encoding for the cytoskeletal anchoring protein, dystrophin, resulting in severe alterations in cytoskeletal structure and impaired muscle morphology. This devastating disease remains incurable, with multiple therapeutic approaches being heavily investigated. Nonetheless, in most instances, this disease is accompanied by prolonged inflammation and fibrosis (Figure 4). Given the role of PAI-1 in modulating the plasminogen system, and thus regulating fibrosis, numerous studies have investigated its role in DMD.

Although PAI-1 is not the primary cause of muscular dystrophy, its atypical expression in resting dystrophic muscle provides some insight into its role. Most studies investigating DMD utilize the *mdx* mouse model. *Serpine1* expression has been shown to dramatically increase in the first 4 months of life in *mdx* mice and begin to decline afterwards [34]. The rapid increase in *Serpine1* is also accompanied by elevations in collagen deposition during early development. Interestingly, fully developed *Serpine1* KO *mdx* animals demonstrated greater fibrosis as a result of increased TGF-β/SMAD signaling, elevated TIMP-1 and Type-I collagen [34]. This was proposed to be a result of excessive elevations in uPA, resulting in the conversion of latent to active TGF-β, and was rescued through the inhibition of uPA. In other words, PAI-1 induced moderate fibrosis as a protective mechanism to reduce the conversion of latent TGF-β.

To complicate matters further, previous work from the same group has shown that *uPA* KO in *mdx* mice has negative consequences particularly as the animals age (i.e., from youth to adulthood), resulting in functionally impaired and chronically degenerating muscle fibres [35]. The lack of uPA reduces the infiltration of immune cells that is needed to clear the degenerating myofibres. To demonstrate the importance of uPA in *mdx* muscle, uPA-derived from bone marrow transplants or the treatment of *mdx* muscle with Ancrod (a fibrinolytic agent) were both shown to promote immune cell infiltration alleviate the chronic degradation of myofibres [35]. In fact, bone marrow transplant experiments were shown to improve the dystrophic phenotype through multiple uPA-dependent mechanisms [35]. Additionally, in vitro studies have shown that PAI-1 and uPAR levels are elevated in primary satellite cells derived from *mdx* muscle [39,145], whereas the aforementioned study suggests otherwise [35]. Nonetheless, the in vitro role of uPA-uPAR-PAI-1 has been implicated for their importance in *mdx* satellite cell survival and migration [39,145]. Together, these data hint at the importance of uPA/uPAR/PAI-1 balance in dystrophic muscle. Imbalances in either of these proteins have negative consequences and exacerbate the dystrophic phenotype and accompanied fibrosis.

### 4.2. Diabetes Mellitus

Diabetes is a disease classified by chronic and abnormal elevation in blood glucose levels. Diabetes affects individuals of all ages and can either be autoimmune (Type 1) or lifestyle-dependent (Type 2). Regardless of the origin, diabetes leads to negative changes to virtually every organ within that body. At the muscle level, diabetes leads to several pathological changes, collectively termed diabetic myopathy, including impaired metabolic capacity, muscle atrophy, reduced capillarization, accumulation of intramyocellular lipids, impaired muscle progenitor cell responses, and elevations in pro-inflammatory markers including PAI-1 [128,146,147,148,149]. Although PAI-1 is a transcriptional target of insulin signaling in multiple tissue/cell types, PAI-1 expression is elevated in Type 1 and 2 diabetes [24,150,151] and in diabetic animal models with no insulin treatment [146]. However, the impact of elevated PAI-1 in diabetes on skeletal muscle health is not well understood. Elevated plasma PAI-1, irrespective of its origin, has been shown to impair the muscle microenvironment, particularly following injury leading to impaired regeneration [27,128] (Figure 4). Most notable from these findings is the fact systemic PAI-1 affected macrophage and satellite cell function following muscle injury [128] and that pharmacologic inhibition of PAI-1 at least partly restores regenerative capacity of both muscle [27] and skin [152]. Future work in this field is highly warranted given the interplay between PAI-1 and skeletal muscle (or other tissues) in diabetic conditions.

### 4.3. Aging

Aging can be characterized by the progressive deterioration of multiple organs and organ systems, leading to mortality. It can be argued that biological aging is a major culprit leading to the development of various diseases. However, it should be noted that aging encompasses multiple hallmarks including genomic instability and loss of proteostasis, resulting in senescence, metabolic dysfunction, altered stem cell function, and other perturbations [153]. These hallmarks have also been associated with PAI-1 expression in numerous age-related diseases [12,32,154]. The role of PAI-1 in aging muscle, however, is largely unknown. Recent work has shown that aged muscle expresses greater levels of PAI-1 following injury [29] (GEO dataset: GDS234; Reference series GSE469), however, it is unclear why this is the case. Aging is associated with elevations in pro-fibrotic, pro-inflammatory, or pro-growth (insulin, IGF-1, etc.) signaling, coupled with oxidative stress that may contribute to elevated PAI-1, yet these findings have not been investigated in aged muscle (Figure 4). It would be of interest to know if aged muscle is a major source of PAI-1 or whether muscle-specific *Serpine1* KO would improve systemic aging. Thus, further investigation of PAI-1 in aging muscle may unravel important features of this protein in maintaining whole-body homeostasis.

## 5. Therapeutic Interventions to Normalize PAI-1

The investigation of PAI-1 is relevant for clinical application to various disease states [16,17,18,19,20,21,22,23,24,25,26,27,28,29,30,31,32,33]. PAI-1 levels are elevated in several diseases states and, in rodent models, the ablation/inhibition of PAI-1 tissue repair [27,28,126,132] whereas deficiency in *Plau* or other components of the plasminogen system has the opposite effect [28,35,37,38,40]. Interestingly, PAI-1 deficiency seems to prolong the lifespan, as demonstrated in transgenic Klotho mice (a model of premature aging) [155], yet the chemical inhibition of PAI-1 does not alter lifespan [156]. Although the clinical goal would be to control PAI-1 activity, PAI-1 deficiency may carry negative consequences. Mutation in human *SERPINE1* is a known cause of a rare bleeding disorder due to poor blood clotting [157,158]. Conversely, heterozygous carriers of a null *SERPINE1* mutation was associated with reduced prevalence of diabetes and an extended lifespan [159]. Thus, it is important to understand the context of which a therapeutic approach/intervention is being utilized. With respect to skeletal muscle, where elevated PAI-1 levels have a negative impact on regeneration and function [27,28,29,34,35,36,37], exercise and pharmacological interventions are among the two common methods for the reduction of PAI-1. This section will shed light on the impact of exercise and pharmacological agents in lowering PAI-1 levels and their relevance in potentially improving skeletal muscle function.

### 5.1. Exercise Approach

Lifestyle choices can influence the expression of PAI-1, and as a result, can lead to negative physiological changes throughout the body. Sedentary behaviour in particular has been shown to negatively impact skeletal muscle regeneration through the aberrant activation of several pathways including inflammatory signaling and leading to fibrosis [160,161,162]. Furthermore, PAI-1 levels tend to be elevated even in healthy sedentary individuals [43]. A strong body of evidence has demonstrated the acute anti-fibrotic response following exercise [44,45,46,47,48,49]. Not only does exercise normalize PAI-1 levels in healthy subjects, but also in diseased populations [50,51,52,53]. Aerobic and resistance exercise have both been shown to influence tPA activity, yet acute resistance exercise may not impact PAI-1 levels to the same extent as aerobic exercise [54,55]. Nonetheless, resistance trained individuals have been found to have lower PAI-1 overall compared to their counterparts [55,56], indicating a potential positive adaptation that ameliorate PAI-1 levels. An interesting point to note is the possibility of acute stress (i.e., damage) to skeletal muscle resulting in transient elevations in PAI-1 as previously shown [29] (GEO dataset: GDS234; Reference series GSE469), yet this response may only be present within skeletal muscle and may be necessary for subsequent adaptations. Nonetheless, it is still unclear whether different intensities or volume of resistance exercise elicit different effects on PAI-1. On the other hand, acute and chronic aerobic exercise elevates tPA levels as high as 180% while reducing PAI-1 activity by almost 40% in an intensity-dependent manner [52,57]. In other words, while moderate intensity aerobic exercise was found to reduce PAI-1 levels and activity in most instances [51,57], high intensity aerobic exercise was shown to continually and significantly reduce PAI-1 levels [43,57,58,59,60]. Regardless of aerobic exercise intensity, levels of tPA remain elevated, while PAI-1 levels remain depressed by 25% for multiple hours post-exercise [51,58]. Moreover, the rigors of exercise and muscle contraction not only improve regeneration [163], but also potentiate the remodeling of the ECM through the modulation of PAI-1 within muscle.

### 5.2. Pharmacological Approach

A number of pleiotropic compounds have been identified to reduce PAI-1 levels/activity. These compounds include metformin, resveratrol, and other antioxidants. A growing body of evidence suggests that these compounds modulate different signaling cascades in distinctive ways. For instance, metformin reduces PAI-1 content by decreasing systemic insulin levels [164,165] or by inhibiting NF-κB by activating adenosine monophosphate-activated protein kinase (AMPK) [166]. Interestingly, metformin has also been shown to protect against muscle damage and improve regeneration [167]. The grape-derived phenol, resveratrol, can elevate autophagy through the activation of AMPK and inhibit TNF/NF-κB/ROS signaling to ultimately diminish PAI-1 levels in a time- and dose-dependent manner [168,169]. At odds with this evidence, resveratrol has also been found to increase TGF-β/SMAD signaling in certain cell types [170,171]. Antioxidants such as curcumin, ginko biloba, shikonin, theaflavin, and others can either reduce PAI-1 indirectly through the reduction of ROS [172,173,174,175,176] or selectively inhibit PAI-1 [61,177]. Indeed, the use of these compounds in skeletal muscle, particularly in damaged or diseased conditions, has not been investigated. Given that most of these compounds are well tolerated with minimum adverse effects and high availability, it may be worth further investigating their role in modulating the ECM through the regulation of PAI-1.

Given the clinical importance of ameliorating PAI-1 levels, numerous selective inhibitors of PAI-1 have been synthesized with some being studied in clinical trials. Most notable of these inhibitors include tiplaxtinin (PAI-039) and the three generations of TM-related drugs (TM5001, TM5009, TM5275, TM5441, TM5509, and TM5614). These inhibitors have been studied in pathological conditions including cancer, cardiovascular disease, pulmonary fibrosis renal diseases, liver diseases and others [17,22,61,62,63,64,65,66,67,68,71]. Most if not all of these inhibitors have been shown to bind the active RCL domain on PAI-1 in a reversible manner to ultimately abolish its ability to bind tPA/uPA [16,69,70]. PAI-039 has also been shown to bind near the somatomedin B domain on vitronectin to inhibit docking of PAI-1 to vitronectin and thereby increasing the likelihood of PAI-1 conversion to its latent conformation [69,70]. In the context of skeletal muscle, these inhibitors have scarcely been used. In fact, only PAI-039 has been used to investigate diabetic muscle regeneration [27], whereas the other inhibitors have not. The use of these inhibitors may provide valuable insight into the role of PAI-1, particularly pertaining to pathological conditions such as muscle dystrophy or aging. Thus, future work investigating PAI-1 should utilize these inhibitors to better understand the importance of this protein in skeletal muscle

## 6. Conclusions

In summary, this review highlights the known and prospective roles of PAI-1 in skeletal muscle with particular emphasis on injury and pathological conditions. This review also provides support for further investigation and testing of different exercise- and drug-based modalities to reduce PAI-1 levels in an attempt to improve skeletal muscle function. Indeed, numerous effects of PAI-1 on cells within muscle tissue (i.e., satellite cells, immune cells, fibrogenic cells, etc.) that have yet to be explored, and as such, future studies on PAI-1 in skeletal muscle should also investigate the implications of this protein on the various cell types found within the skeletal muscle tissue. Investigation in this manner may provide further insight into the role of PAI-1 in modulating the aberrant changes observed in skeletal muscle tissue and may provide novel insight into safety and efficacy of therapeutic approaches.

## Abbreviations

AMPK	Adenosine monophosphate-activated protein kinase
AP1	Activation protein 1
ATP	Adenosine triphosphate
cAMP	Cyclic adenosine monophosphate
CREB1	cAMP-responsible element binding protein 1
ECM	Extracellular matrix
FDP	Fibrin degradation product
GC	Glucocorticoid
GSK3	Glycogen synthase kinase 3
hD/E/F	Helix D/E/F domains
HIF	Hypoxia-inducing factor
IGF-1	Insulin-like growth factor-1
IL	Interleukin
LRP1	Low density lipoprotein receptor-related protein 1
MAPK	Mitogen-activated protein kinase
MMP	Matrix metalloproteinase
NF-κB	Nuclear factor kappa B
PAI-1	Plasminogen activator inhibitor-1
PI3K	Phosphatidylinositol 3-kinase
PKA	Protein kinase A
RCL	Reactive center loop
ROS	Reactive oxygen species
TGF-β	Transforming growth factor-β
TIMP	Tissue inhibitor of metalloproteinase
TNF	Tumor necrosis factor
tPA	Tissue-type plasminogen activator
uPA	Urokinase-type plasminogen activator
uPAR	Urokinase-type plasminogen activator receptor
